# Autosomal recessive *ELOVL1*-related disorder presenting with severe neonatal cholestasis: A novel clinical feature?

**DOI:** 10.1016/j.ymgmr.2026.101305

**Published:** 2026-03-11

**Authors:** Anne Chun-Hui Tsai, Hsuan-Tung Lee, Bianca Sanchez, Yu-Ren Huang, Sarah Krysinski, Alice Zalan, Erin Falsey

**Affiliations:** a Division of Genetics, Department of Clinical Pediatrics, University of Illinois Chicago, 840 S. Wood Street, Chicago, IL 60612, USA.; b College of Medicine, Taipei Medical University, No. 250 Wuxing Street, Taipei City 11031, Taiwan.; c Department of Clinical Pediatrics, University of Colorado, 80045, USA; d Department of Genetics and Genomic Sciences, Case Western Reserve University and University Hospitals, Cleveland, OH 44106, USA

**Keywords:** ELOVL1-related disorders, Neonatal cholestasis, Very-long-chain fatty acids, Bile acid metabolism, Cholic acid therapy

## Abstract

ELOVL1-related disorders are typically characterized by neurological and dermatological features. We present a novel case of severe neonatal cholestasis in a female neonate with autosomal recessive inheritance with a homozygous variant in *ELOVL1* (c.458G > A; p.Trp153*). The patient demonstrated direct hyperbilirubinemia, low levels of very-long-chain fatty acids (VLCFA), a newborn screen C26 level of 0, and severe cholestasis. Treatment with ursodeoxycholic acid worsened the patient's cholestasis, whereas cholic acid supplementation resulted in significant clinical and biochemical improvement. The ichthyosis resolved with optimal nutrition and topical application of coconut oil moisturizer. This report expands the known phenotypic spectrum of ELOVL1-related disorders to include primary liver dysfunction.

## 1 Introduction

*ELOVL1* (elongation of very-long-chain fatty acids 1) encodes an enzyme that catalyzes the elongation of saturated and monounsaturated fatty acids into very-long-chain fatty acids (VLCFAs), which are integral to cellular membrane structure and bioactive lipid molecules [1]. Pathogenic variants in *ELOVL1* have been associated with a phenotypic spectrum involving neurological and dermatological abnormalities, including ichthyotic keratoderma, spasticity, hypomyelination, and dysmorphic features [2]. Both autosomal dominant (AD) and autosomal recessive (AR) inheritance have been described [2], [3], [4], [5], [6].

VLCFAs and their derivatives, such as sphingolipids, are crucial for maintaining cellular membrane integrity, facilitating cellular signaling, and contributing to bile composition [7]. Loss of ELOVL1 enzymatic function impairs VLCFA biosynthesis, potentially disrupting multiple biochemical pathways and organ systems. To date, hepatic involvement–specifically neonatal cholestasis–has not been reported in association with ELOVL1-related disorders. This patient with autosomal recessive ELOVL1 deficiency presented with severe neonatal cholestasis, which responded well to cholic acid therapy, thereby broadening the phenotypic spectrum of ELOVL1-related disorders to include primary hepatic dysfunction.

## 2 Case presentation

**Fig. 1:**
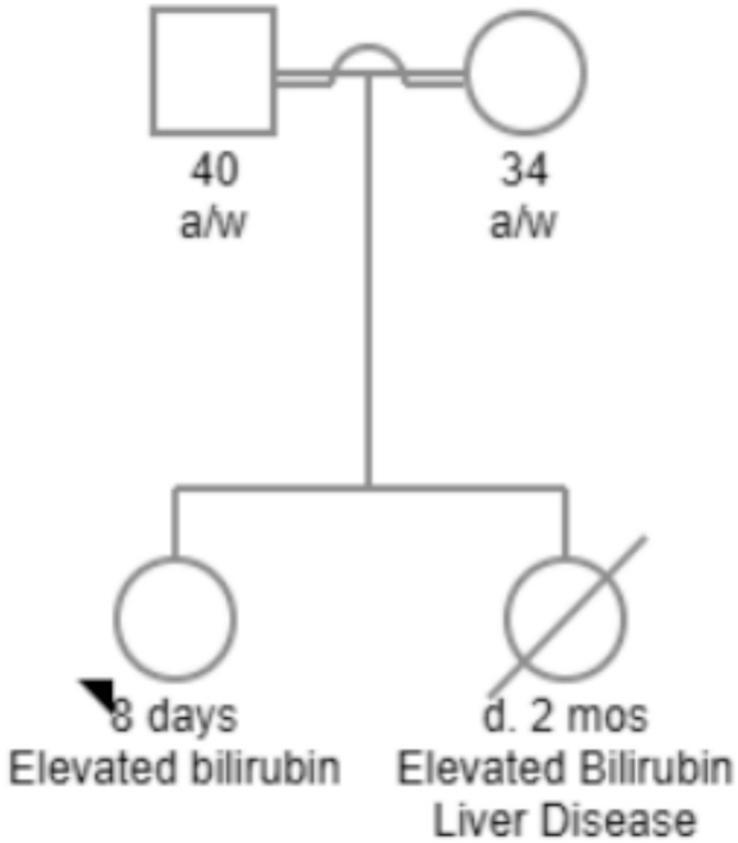
Pedigree of first-degree relative. Pedigree showing parental consanguinity and a deceased female sibling who had elevated bilirubin and liver disease, resulting in death at 2 months of age.

The proband is the second child born to a consanguineous couple (uncle-niece relationship). Both parents are phenotypically normal. She was delivered via cesarean section at 38 weeks of gestation due to non-reassuring fetal heart tones to her 34-year-old mother (gravida 2, para 0–1–0-0). Apgar scores were 8 and 9 at 1 min and 5 min, respectively. The family history was notable for a deceased female sibling who developed severe hyperbilirubinemia and died of liver disease at 2 months of age (pedigree in Fig. 1).

**Fig. 2:**
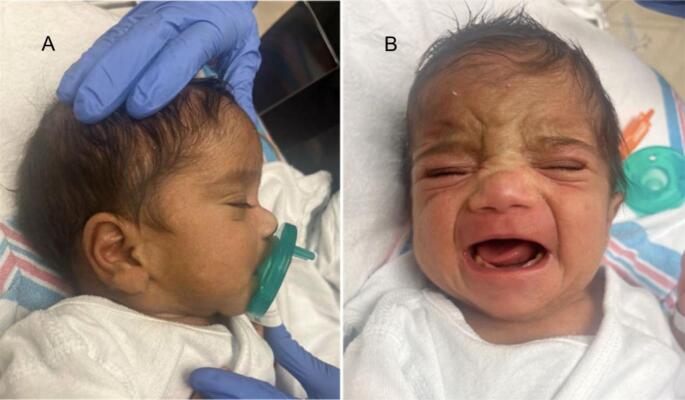
Photograph of the proband showing generalized jaundice. The image was taken at day 8 of life, during the initial presentation with direct hyperbilirubinemia. Diffuse jaundice is seen.

On day 8 of life, the patient was admitted to the neonatal intensive care unit (NICU) for evaluation of an abnormal newborn screen and conjugated hyperbilirubinemia (total bilirubin: 16 mg/dL; direct bilirubin: 5 mg/dL), consistent with neonatal cholestasis (patient photo shown in Fig. 2). Initial metabolic evaluation, including a total lipid and VLCFA assay, revealed undetectable C26:0, low levels of C24:0, C22:0, and phytanic acid, as well as a decreased C24:C22 ratio. These findings, combined with the unexplained cholestasis, prompted further investigation. Abdominal ultrasound, cholangiogram, and magnetic resonance cholangiopancreatography revealed no portal hypertension or structural abnormalities of the hepatobiliary system. Liver biopsy demonstrated cholestasis, scattered apoptotic hepatocytes, and mild fibrosis of the portal tracts, with no histologic features of biliary atresia. Additional clinical findings included failed newborn hearing screening, bilateral myopia, nystagmus and recurrent *Salmonella*-associated gastroenteritis and enteric fever.

### 2.1 Molecular analysis

A single-nucleotide polymorphism (SNP)-based chromosomal microarray (CMA) revealed no copy number variations but identified regions of homozygosity (ROH) encompassing 194.5 Mb (approximating 7% of the genome), consistent with consanguinity, uncle and niece. Whole exome sequencing (WES) identified a homozygous variant in *ELOVL1* (c.458G > A, p.Trp153*), which is associated with ELOVL1-related disorder and is concordant with the patient's VLFCA profile. Based on the biochemical, clinical, and genetic data, the variant was interpreted as pathogenic. WES also identified a homozygous pathogenic variant in *COL9A2* (c.1051C > T), associated with Stickler syndrome, and compound heterozygous variants in *SPTB* (c.2386C > T and c.2333C > T), linked to hereditary spherocytosis and elliptocytosis (Table 1). While the *COL9A2* and *SPTB* variants are notable findings, they are unlikely to account for the primary hepatic presentation of severe cholestasis.

**Table 1:** Genetic findings from WES in the proband.

Gene (variant)	Zygosity	Condition
*ELOVL1* (c.458G > A)	Homozygous	ELOVL1-related disorder
*COL9A2* (c.1051C > T)	Homozygous	Stickler syndrome
*SPTB* (c.2386C > T and c.2333C > T)	Compound heterozygous	Hereditary spherocytosis and elliptocytosis

Initial treatment included supplementation with fat-soluble vitamins, docosahexaenoic acid (DHA), and ursodeoxycholic acid. However, cholestasis worsened despite therapy, with total bilirubin rising to 46 mg/dL and direct bilirubin to 35 mg/dL by 5 months of age; AST/ALT were elevated at 208 U/L and 115 U/L and peaked at 444 U/L and 555 U/L, respectively; ALP was markedly elevated at 1063 U/L when cholestasis developed and peaked at 1396 U/L; GGT was initially normal (50 U/L) when cholestasis developed, but markedly increased from 118 U/L to a peak of 684 (U/L) after starting ursodeoxycholic acid. Serum albumin and INR remained normal. Urinary bile acid analysis demonstrated elevated excretion, suggestive of a bile acid synthesis defect. Due to the clinical deterioration, the patient was listed for liver transplantation. Ursodeoxycholic acid was subsequently discontinued and replaced with cholic acid. Following this change, bilirubin levels improved from 46/27 mg/dL to 26/17 mg/dL within 2 months; ALP decreased to 675 U/L within 3 months; GGT remained elevated despite improvement of cholestasis but gradually decreased from 684 U/L to 377 U/L, and eventually normalized to 57 U/L after 6 months. Continued treatment with cholic acid led to progressive normalization of bilirubin levels and complete resolution of cholestasis. The trends of bilirubin, GGT, AST, ALT, and C26 levels are provided in Table 2. Consequently, the patient was removed from the liver transplant list.

**Table 2:** Trends of bilirubin, GGT, AST, ALT, and C26.

Date	Age (months)	Total bilirubin (mg/dL)	Direct bilirubin (mg/dL)	GGT (U/L)	AST (U/L)	ALT (U/L)	C26 (μg/mL)
12/18/2023	0.9	–	–	107	–	–	Undetectable at newborn screening
01/18/2024	1.9	10.5	6.5	–	196	99	
01/21/2024	2.0	12.6	7.6	–	243	112	
02/02/2024	2.4	–	–	55	–	–	
02/03/2024	2.4	16.5	9.4	–	208	115	
02/08/2024	2.6	–	–	61	–	–	
02/21/2024	3.0	–	–	60	–	–	
04/22/2024	5.0	46	35	–	–	–	
05/28/2024	6.2	–	–	118	–	–	
06/10/2024	–	–	–	–	–	–	0.05 (reference: 0.23±0.09)
06/30/2024	7.3	–	–	346	–	–	
07/09/2024	7.6	–	–	572	–	–	
07/12/2024	7.7	–	–	647	–	–	
08/06/2024	8.5	0.9	0.5	684	444	555	
09/03/2024	9.4	–	–	377	–	–	
11/22/2024	12.0	–	–	57	–	–	
12/19/2024	–	–	–	–	–	–	0.07
03/11/2025	15.6	–	–	22	–	–	
05/05/2025	–	–	–	–	–	–	0.17
06/17/2025	18.8	–	–	17	–	–	
11/10/2025	23.6	0.4	–	–	73	69	

**Fig. 3:**
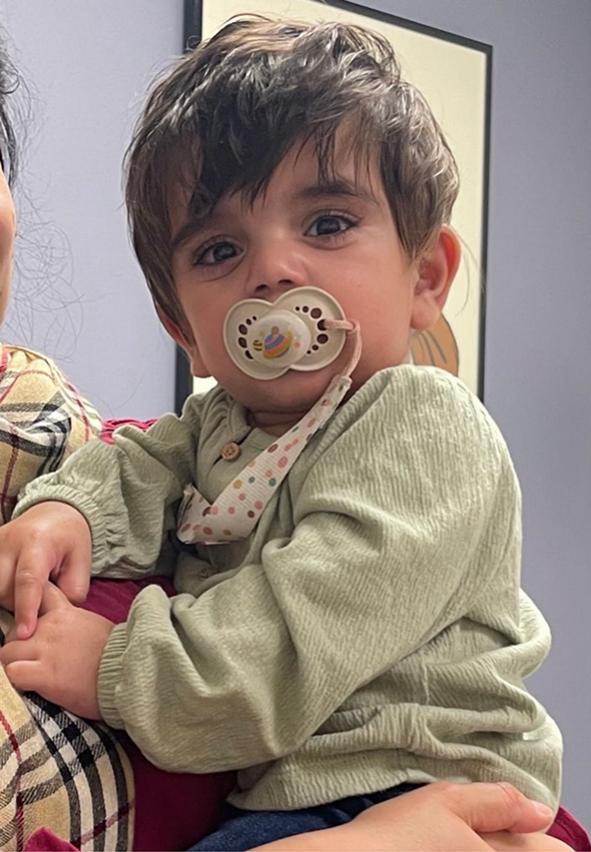
Photograph of the proband at 12 months of age. The image shows the proband without jaundice and in good general health. No significant skin lesions are visible.

At the 18-month follow-up, the patient exhibited complete resolution of jaundice, stable weight gain, and achievement of developmental milestones, including independent sitting, social engagement, and early verbalization. However, mild global delays were observed. Bilirubin levels were within normal limits (total: 0.3 mg/dL; direct: 0.1 mg/dL). Repeated plasma VLCFA testing showed normal C26:0 (0.17 μg/mL) with a low C24/C22 ratio (0.53) and a normal C26/C22 ratio (0.010). Ongoing management included DHA supplementation and dietary introduction of foods enriched in C24 and C26 fatty acids. The patient's skin showed no significant lesions, and mild dryness was effectively managed with coconut oil (photo shown in Fig. 3). Her myopia due to known Stickler syndrome, horizontal pendular nystagmus, heart defects (pulmonic stenosis and aortic regurgitation), and hematological status remained stable.

## 3 Discussion

This report describes a novel presentation of ELOVL1-related disorder with severe neonatal cholestasis as the primary clinical manifestation. To the best of our knowledge, this is the first documented case of ELOVL1-related disorder manifesting as primary hepatic dysfunction.

Notably, the cholestasis worsened with ursodeoxycholic acid therapy but responded well to cholic acid, suggesting a potential bile acid synthesis defect that may be exacerbated by ursodeoxycholic acid [8]. Parental consanguinity and a sibling that died at 2 months due to similar presentation (jaundice and liver failure requiring a transplant in Pakistan) raise the likelihood of other AR conditions; however, WES did not reveal an alternative genetic diagnosis that better explains the cholestasis. These findings raise the possibility that impaired VLCFA elongation due to ELOVL1 dysfunction may contribute to cholestasis by disrupting hepatocellular membrane composition and bile acid transport mechanisms.

**Fig. 4:**
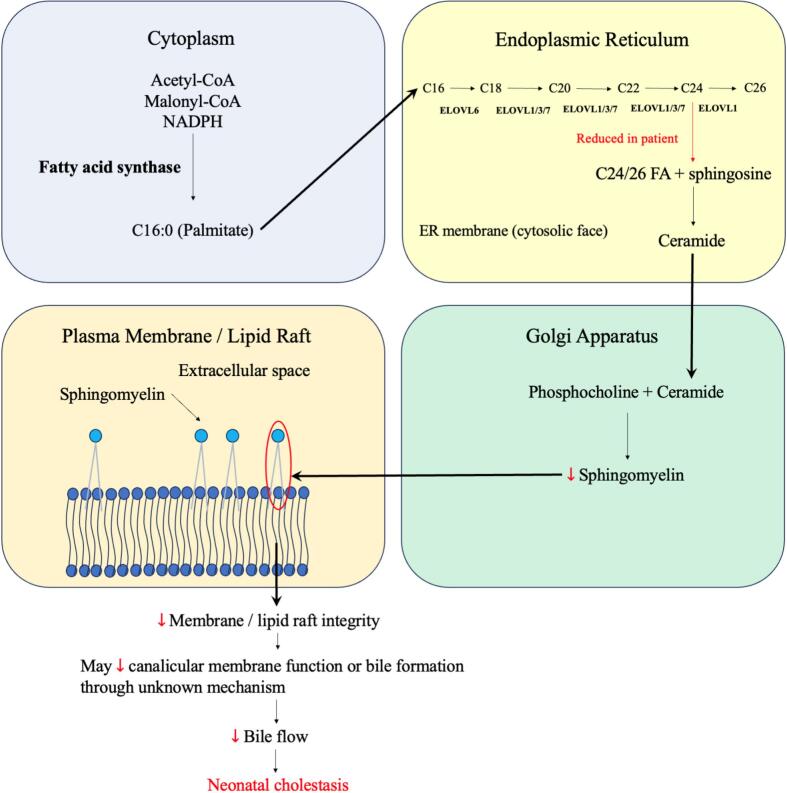
Schematic of sphingomyelin synthesis and its disruption in ELOVL1 deficiency. Palmitate (C16:0) synthesized in the cytoplasm is elongated at the ER by the ELOVL enzymes. ELOVL1 catalyzes the final steps to generate C24/26 fatty acids, which are used to synthesize sphingomyelin in the Golgi apparatus. Sphingomyelin integrates into the outer leaflets of plasma membrane and lipid rafts. In patients with ELOVL1 deficiency, reduced C24/26 may lead to sphingomyelin deficiency, cellular membrane disruption, and impair bile formation through an unknown mechanism, contributing to neonatal cholestasis.

ELOVL1 is a key member of the VLCFA elongase superfamily, which consists of enzymes localized to the endoplasmic reticulum. These enzymes facilitate the elongation of fatty acids by adding two carbon units to the acyl chain during fatty acid synthesis [9]. Specifically, ELOVL1 demonstrates activity towards saturated and monounsaturated fatty acids, particularly C20-CoA and C22-CoA and is essential for the biosynthesis of C24-sphingolipids such as ceramides and glycosphingolipids [10]. These lipids are critical in cellular signaling, metabolism, and formation of the lipid bilayer [7]. Although ELOVL1 expression is prominent in skin and neural tissues, it is also present in hepatocytes, where VLCFAs contribute to membrane composition and can influence lipid raft organizations [9], [11], [12], [13], [14], [15]. Defective VLCFA elongation may compromise the integrity of hepatocyte membranes and thereby predispose to cholestasis, although the precise mechanism remains uncertain. Lipid rafts, enriched in sphingolipids and cholesterol, regulate the localization and function of membrane proteins involved in bile excretion and formation [16], [17], [18]. Disruption of lipid raft composition due to VLCFA deficiency may impair these pathways (Fig. 4). In a previous study, lipidomic analyses revealed decreased C24:0/C22:0 and C26:0/C22:0 ceramide and sphingomyelin ratios in patient keratinocytes and fibroblasts, suggesting that these lipid ratios could serve as potential biomarkers of disease activity and therapeutic response in ELOVL1-related disorders [3].

**Fig. 5:**
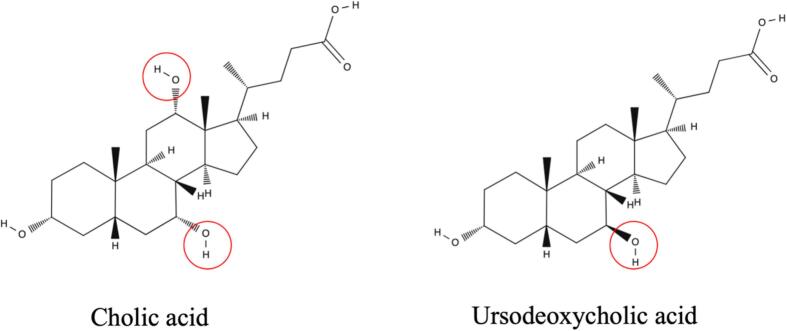
Cholic acid and ursodeoxycholic acid. Cholic acid (trihydroxy) and ursodeoxycholic acid (dihydroxy) have different number of hydroxy groups and different stereochemistry. Note the 7β-hydroxyl group circled in ursodeoxycholic acid.

Furthermore, ELOVL1 has been shown to modulate liver X receptor (LXR) activity, a central regulator of bile acid synthesis and cholesterol homeostasis [9]. Dysfunction of ELOVL1 may disrupt LXR-mediated pathways, leading to aberrant bile acid metabolism. The paradoxical worsening of cholestasis with ursodeoxycholic acid remains unexplained; we speculate that altered membrane composition and/or disrupted LXR-mediated pathways could have contributed [19]. Ursodeoxycholic acid is a 7 beta-hydroxy epimer of chenodeoxycholic acid, one of the primary bile acid end products synthesized alongside cholic acid in the liver (Fig. 5). Cholic acid, the other end product of the bile synthesis pathway, bypasses endogenous synthetic pathways and functions as a primary bile acid replacement. It replaces deficient products in bile acid synthesis defects, restores bile excretion, and protects hepatocytes from further damage [20], [21]. In our patient, the contrasting responses to ursodeoxycholic acid and cholic acid suggest that bile acid-directed therapy may be relevant, although the underlying mechanism of this difference remains uncertain.

The patient's concurrent *Salmonella* infection, which caused enteric fever, may have also contributed to the severity of cholestasis. Systemic infections are known to exacerbate liver dysfunction by inducing inflammatory responses and impairing bile flow [22]. This may hinder the efficient elimination of certain pathogens, such as *Salmonella*, allowing persistent colonization and biofilm formation [[23], [24]]. The persistent elevation and slow improvement of GGT despite improvement in bilirubin may suggest ongoing cholangiocyte-related injury, potentially influenced by the infection, although the exact mechanism remains unclear. The synergistic effects of cholestasis associated with ELOVL1-related disease and *Salmonella* infection may have contributed to the clinical presentation in this patient. Her AST/ALT essentially normalized at 1 year of age upon being removed from the transplant list. However, the levels remained slightly elevated at around 50 U/L, which might be secondary to cholic acid supplementation.

ELOVL1-related disorders have been reported with both AD and AR inheritance patterns (Table 3 is a comparison of our patient and reported cases). Three previous cases with AD inheritance are identified, all of which involve heterozygous c.494C > T variants [2], [3], [4]. These patients presented with ichthyosis, spasticity, hypomyelination, nystagmus, and bilateral sensorineural hearing loss, with disease progression since birth or infancy. The study by Mueller et al. suggests that the c.494C > T variant exerts a dominant-negative effect, whereby the mutant ELOVL1 protein interferes with the function of the wild-type protein within the elongase complex, thereby reducing overall enzyme activity despite heterozygosity [3].

**Table 3.** *ELOVL1* variants and clinical features in reported cases.

Author	Genotype	Parental relationship	Inheritance pattern	Ethnicity	Age of onset	Neurologic and sensory	Dermatologic	Biochemical findings	Other systemic involvement or imaging findings
Present study	c.458G > A, homozygous	Consanguineous, uncle and niece	AR	Pakistani	Identified via NBS	Failed neonatal hearing test, bilateral myopia	Dry skin	Undetectable C26:0 on NBS, with low C24:0, C22:0, phytanic acid, and C24/C22	Neonatal cholestasis
Kutkowska-Kazmierczak et al., 2018. Mueller et al., 2019	Patient 1, c.494C > T, heterozygous	Non-consanguineous	AD	Polish	Skin issue and slowing of psychomotor development started at 7 months	Spastic paraplegia, ataxia, narrowing of the visual field, progressive rotatory nystagmus, astigmatism (−5 dpt), bilateral high frequency SNHL	Ichthyosis	Normal individual VLCFA levels; low C24/C22 ratio	Mild dysmorphic facial features, mild hypomyelination on MRI
Kutkowska-Kazmierczak et al., 2018. Mueller et al., 2019	Patient 2, c.494C > T, heterozygous	Non-consanguineous	AD	Polish	Skin issue started since early infancy; signs of spasticity started within the first year of life	Spastic paraplegia, ataxia, rotatory nystagmus, astigmatism (−4.5 dpt), bilateral high frequency SNHL	Ichthyosis	Normal individual VLCFA levels; low C24/C22 ratio	Mild dysmorphic facial features, mild hypomyelination on MRI
Takahashi et al., 2022	Patient 3 (sibling of patient 4), c.376-2 A > G, homozygous	Consanguineous, first-degree cousins	AR	Turkish	Gradual progression since early infancy	Spastic paraplegia, ataxia, tremor, horizontal nystagmus, high frequency SNHL	Ichthyosis, hyperkeratosis	Decreased acylceramides; normal protein-bound ceramides; decreased ceramides containing FAs with 25 carbons or more	Dysmorphic facial features, hypomyelination on MRI
Takahashi et al., 2022	Patient 4 (sibling of patient 3), c.376-2 A > G, homozygous	Consanguineous, first-degree cousins	AR	Turkish	Gradual progression since early infancy	Spastic paraplegia, ataxia, tremor, horizontal nystagmus, high frequency SNHL	Ichthyosis, hyperkeratosis	Decreased acylceramides; normal protein-bound ceramides; decreased ceramides containing FAs with 25 carbons or more	Dysmorphic facial features, hypomyelination on MRI
Marcoux et al., 2025	c.494C > T, heterozygous	Non-consanguineous	AD	French Canadian	Dry skin noted at birth, global developmental delay present since early infancy	Spasticity, hyperreflexia, ataxia, tremors, horizontal nystagmus, astigmatism, bilateral SNHL	Ichthyosis, severe pruritus	Low C24/C22 ratio	Moderate intellectual disability, hypomyelinating leukodystrophy, small optic tracts
Wong et al., 2025	Seven cases reported. Case 1, c.462G > A; cases 2–3 (siblings), c.248C > T; case 4, c.457 T > C; case 5, c.491G > A; case 6–7 (siblings), c.52C > T. All homozygous	Consanguineous	AR	Not reported	Not precisely reported; congenital ichthyosis in 5/7; neurodevelopmental delay described in infancy; spasticity progressive (onset age not specified)	Motor delay in all; all can sit by 16 months; walking achieved in 3; loss of walking in 1. Movement disorder: axial hypotonia (6/6), head tremor (7/7), dystonia (4/7), myoclonus (6/7), dysarthria (6/6). Photophobia in 2, nystagmus (5/6)	Congenital ichthyosis in 5/7	Plasma VLCFA analysis only in case 1, showed decreased C24:0 and C26:0	Mild intellectual disability in 4/5, hypomyelinating leukodystrophy (6/6), cerebellar involvement, hypoplasia of corpus callosum (5/6)

Abbreviations: AD, autosomal dominant; AR, autosomal recessive; dpt, dioptres; ELOVL1, elongation of very-long-chain fatty acids 1; FA, fatty acid; MRI, magnetic resonance imaging; NBS, newborn screening; SNHL, sensorineural hearing loss; VLCFA, very-long-chain fatty acid.

In contrast, Takahashi et al. reported AR inheritance in a pair of siblings with a homozygous c.376-2 A > G splice site mutation, who presented with phenotypes similar to the AD cases [5]. More recently, Wong et al. reported seven individuals with biallelic *ELOVL1* variants [6]. The predominant phenotype included ichthyosis (5/7), developmental delay (7/7), progressive spasticity (7/7), a complex movement disorder with head tremor (7/7) and myoclonus (6/7), and non-progressive hypomyelination on brain MRI. Notably, hearing impairment was not found in their cohort. In the present case with homozygous c.458G > A variant, recessive inheritance likely resulted in a more functional loss of the ELOVL1 protein, contributing to the severe and early onset hepatic phenotype. The absence of hepatic involvement in previously reported cases may also reflect underlying genotypic and environmental variability. It should be noted that this proband also carried a diagnosis of Stickler syndrome, which may have contributed to the bilateral myopia and failed newborn hearing screening. In addition, this case reflects a genomic medicine approach in which early diagnosis and management, such as supplementation of food enriched in C24 and C26 fatty acids, may modify or prevent the classic phenotype. Neurologic features (spasticity, myoclonus) have not yet been documented in our patient, and continued follow-up is required to determine the neurologic trajectory. An elective brain MRI for baseline is scheduled. Expanded reporting of *ELOVL1* variants across different clinical presentations will enhance the genotype-phenotype correlations and improve the understanding of organ-specific manifestations.

## 4 Conclusion

This report expands on the phenotypic spectrum of ELOVL1-related disorders to include neonatal cholestasis. The patient's contrasting response to ursodeoxycholic acid and cholic acid underscores the importance of individualized, bile acid-directed therapy enabled by early genomic diagnosis, while the underlying mechanism remains unclear. These findings suggest that ELOVL1 dysfunction may impair hepatocellular excretion of bile acid through undefined pathways. Further studies are needed to delineate variant-specific organ involvements, clarify genotype-phenotype correlations, and improve targeted treatment strategies.

## CRediT authorship contribution statement

**Anne Chun-Hui Tsai:** Writing – review & editing, Writing – original draft, Validation, Supervision, Investigation, Conceptualization. **Hsuan-Tung Lee:** Writing – original draft, Visualization, Investigation. **Bianca Sanchez:** Writing – review & editing, Investigation, Conceptualization. **Yu-Ren Huang:** Writing – review & editing, Investigation, Conceptualization. **Sarah Krysinski:** Writing – review & editing, Investigation, Conceptualization. **Alice Zalan:** Writing – review & editing, Investigation, Conceptualization. **Erin Falsey:** Writing – review & editing, Investigation, Conceptualization.

## Funding

This research did not receive any specific grant from funding agencies in the public, commercial, or not-for-profit sectors.

## Declaration of competing interest

The authors declare that they have no known competing financial interests or personal relationships that could have appeared to influence the work reported in this paper.

## Data Availability

Data will be made available on request.
